# Unexpected Relations of Historical Anthrax Strain

**DOI:** 10.1128/mBio.00440-17

**Published:** 2017-04-25

**Authors:** M. H. Antwerpen, J. W. Sahl, D. Birdsell, T. Pearson, M. J. Pearce, C. Redmond, H. Meyer, P. S. Keim

**Affiliations:** a Bundeswehr Institute of Microbiology, Munich, Germany; b The Pathogen and Microbiome Institute, Northern Arizona University, Flagstaff, Arizona, USA; c Defence Science and Technology Laboratory, Porton Down, United Kingdom; The Sanger Institute

## Abstract

In 1998, it was claimed that an 80-year-old glass tube intentionally filled with *Bacillus anthracis* and embedded in a sugar lump as a WWI biological weapon still contained viable spores. Today, genome sequencing of three colonies isolated in 1998 and subjected to phylogenetic analysis surprisingly identified a well-known *B. anthracis* reference strain isolated in the United States in 1981, pointing to accidental laboratory contamination.

## IMPORTANCE

Next-generation sequencing and subsequent phylogenetic analyses are useful and reliable tools for the classification of recent and historical samples. The reliability of sequences obtained and bioinformatic algorithms has increased in recent years, and research has uncovered the identity of a presumed bioweapon agent as a contaminant.

## OBSERVATION

The recent rapid development of next-generation sequencing (NGS) technologies combined with bioinformatics provides useful tools for reliable microbial isolate identification down to the strain level. For comparative genomic investigations, NGS leads to a better understanding of the epidemiology and evolution of various microbial organisms. Sequencing of historical specimens represents previously unattainable evolutionary data. Thus, contemporary emerging isolates are sequenced (1) and so are historical specimens (e.g., *Yersinia pestis* [2] from teeth of victims of the Justinian plague or *Variola major* [3] from a 400-year-old mummy).

The oldest isolate of *Bacillus anthracis*, the causative agent of anthrax, dates back to 1917 (4). The German spy Baron Otto von Rosen was imprisoned in 1917 in Karasjok, Norway, near the current Finnish border and accused of smuggling a biological weapon consisting of anthrax-filled glass capillaries hidden in sugar lumps with the intent to sabotage the transportation lines that relied upon reindeer during the Great War (5). The confiscated sugar lumps were stored for 8 decades at the police museum in Trondheim, Norway, before “rediscovery” and sent to the Defence Evaluation Research Agency, Chemical and Biological Defence, in Porton Down, United Kingdom, in 1997 (4). Direct PCR of the vial contents with species-specific primers identified the presence of *B. anthracis* DNA. Following extensive culturing efforts, four bacterial colonies were isolated. By using *B. anthracis*-specific PCR assays targeting sequences of the chromosome and both *B. anthracis*-specific plasmids, the identity of the colonies as *B. anthracis* was confirmed and published, emphasizing the sturdiness of spores (4) having survived more than 80 years at ambient temperature.

In this study, we reinvestigated three of the four colonies by NGS with the intent to subtype and characterize a 100-year-old *B. anthracis* strain. The DNAs were sequenced independently, and this resulted in three draft genomes (~5.5 Mb). The genomes of strains sugar 2 and sugar 4 were identical to each other and differed from that of strain sugar 3 by a single nucleotide polymorphism (SNP) at position 883096 (C → T). In a direct alignment of the three sugar draft genomes with that of the Ames “ancestor,” there were 5,474,730 high-quality nucleotide positions available for genotypic comparison.

The initial molecular subtyping by canonical SNP typing (6, 7) assigned the strains to the A.Br.Ames clade. Multilocus variable-number tandem-repeat analysis (MLVA) with two different established systems (6, 8) revealed the same allelic patterns as for the Ames ancestor strain. Subsequent *in silico* analysis confirmed the laboratory results. Further *in silico* investigations by whole-genome SNP typing revealed only two nonsynonymous SNPs, at positions 1798709 (A → G) and 4212867 (G → A) compared to the Ames ancestor. No difference was detected in the virulence plasmids, whose complete sequences were also determined. This high identity was unexpected, as the Ames ancestor strain was isolated from a cow in Sarita, TX, in 1981, 64 years after the historical sample’s discovery (9). Extensive research after the 2001 Amerithrax letter attacks showed that members of the A.Br.Ames clade were naturally uncommon (10). Phylogeographic analysis was consistent with the derived Ames clade’s historical importation into North America, perhaps from China, where many close relatives are found (11).

In particular, the Ames ancestor and its identical sister genome, FTD1004, are very high quality closed and finished sequences that represent two stocks derived directly from the original 1981 Texas *B. anthracis* isolate. These two stocks were independently established in May 1981 after material was transferred to USAMRIID. Together, they represent the oldest known laboratory stocks of the original Ames strain, established within months of the bovine anthrax case.

**FIG 1:**
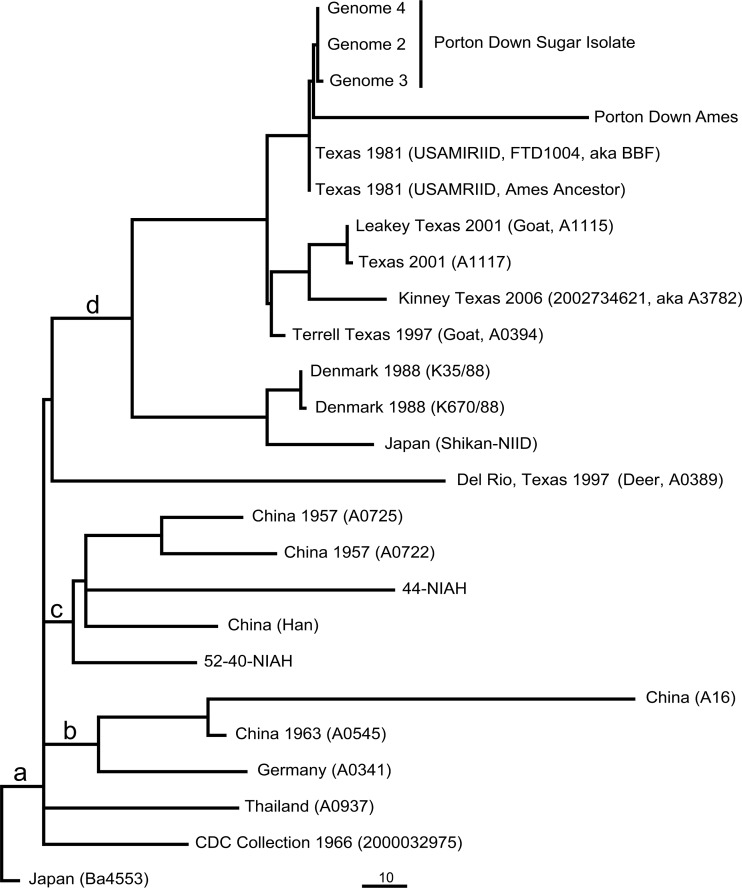
High-resolution phylogeny of the Ames clade of *B. anthracis*. A whole-genome phylogeny based upon 25 high-quality draft or completely finished *B. anthracis* genomes is shown. A total of 812 SNPs, with no missing data, were used to construct a maximum-parsimony tree with a consistency index of 1.0. Branches previously identified and named (16) are labeled a (A.Br.081), b (A.Br.085), c (A.Br088), and d (A.Br.001). For the SNP genotypes used to construct the phylogeny shown, see Table S1 in the supplemental material.

Directly diverging from these five genomes (Fig. 1) is that of strain Porton Down Ames, with 65 unique SNPs. Careful examination of the data argues that these are not sequencing errors but rather represent mutations that occurred during laboratory growth. A similar divergence from other Ames isolates was reported by Read et al. and attributed to mutations that occurred during a plasmid-curing regimen (12, 13).

10.1128/mBio.00440-17.1 TABLE S1  SNP matrix for the Ames strain and close relatives. Shown is a spreadsheet containing the SNP genotypes used to construct the phylogeny shown in Fig. 1. The Ames ancestor genome (AE017334.2) positions are presented in columns 1 and 2, while the remaining columns contain the SNP genotypes of individual genomes. Download TABLE S1, XLSX file, 0.1 MB. Copyright © 2017 Antwerpen et al. 2017 Antwerpen et al. This content is distributed under the terms of the Creative Commons Attribution 4.0 International license.

We can only speculate about the direct linkage between the original Texas 1981 Ames strains and the Porton Down sugar isolates of 1998. The Porton Down scientists were the world leaders in anthrax research, and their plasmid-cured Ames strain is still in use in reference laboratories worldwide. Therefore, they were well aware of the value of this historical sample and fumigated the class 3 microbiological safety cabinet with formaldehyde prior to opening the vials to avoid contamination (4). These careful protocols were invoked to cultivate these historical specimens, which would lead to the oldest *B. anthracis* strains ever described, as previously the oldest samples were from 1954 (14).

It is clear from their study description that the isolation process was more difficult than the standard microbiological methods employed for *B. anthracis* (4). Because no colonies were apparent from culturing of the original liquid, an 8-day enrichment in liquid broth was used. Plating of the enriched liquid resulted in only four colonies, which were confirmed to be *B. anthracis* by McFadyean’s test and species-specific PCR assays. However, none of these techniques was able to differentiate among *B. anthracis* strains. The first suitable molecular typing techniques (MLVA) using eight markers was published in 2000 (15), 2 years later, and hence, molecular typing of the isolates was indeed not possible at the time of the report.

Despite secure handling and microbiological safety cabinet cleaning, it seems highly likely that the isolated strains are accidentally isolated contaminants of formerly processed Ames strains. In 1981, the progenitor of the Ames ancestor and FTD1004 was isolated in Texas and then transferred to USAMRIID at Ft. Detrick, MD, United States. In 1982, an Ames culture was transferred from Ft. Detrick to Porton Down (12). Only one phylogenetically informative SNP separates the Porton Down genomes from the USAMRIID Ames genomes (Fig. 1). This high identity and phylogenetic topology argue that the Porton Down Ames strain and Porton Down sugar contaminants were derived from the Ames material sent in 1982.

In the context of spatial separation of analytical and research facilities to avoid contamination, there were no dedicated analytical facilities at the Porton Down defense laboratories in 1998. Therefore, analysis of the sample had to be conducted in a facility used for research activities. This changed in 2006, when the Defence Science and Technology Laboratory implemented a purpose-built reception and screening facility. Culturing of samples is now carried out in a separate facility to mitigate against the possibility of laboratory contamination.

When genome diversity is so low that only a few SNPs differentiate critical isolates, high-quality NGS and careful phylogenetic analyses are needed for unambiguous strain identification. Only this ultradiscriminatory power has unraveled the mystery of the historical *B. anthracis* sugar lumps, revealing that the spores really did not survive for this extended period. Today, direct metagenomic analysis of the capillary fluid might be capable of generating the whole genome sequence, even if there were no viable spores (16). The investigation of historical pathogen specimens has clearly entered a new era.

### Materials and methods.

All strains (sugar 2, sugar 3, and sugar 4) were successfully grown on sheep blood agar out of deep-frozen cryobank stocks in a biosafety level 3 (BSL3) area. Strain sugar 1 failed to grow. After incubation overnight at 37°C and visual inspection for contamination, single colonies were picked and diluted. Subsequently, the suspension was filtered with a Millipore 0.1-µm filter to obtain spore-free liquids and its nucleic acid was purified with the Qiagen DNA Blood and Tissue kit (Qiagen, Hilden, Germany). In accordance with standard procedures, 10% of the volume was plated again onto sheep blood agar and incubated for 14 days without any growth characteristics, prior to transfer of the DNA out of the BSL3 laboratory. Final DNA concentrations were measured with the Qubit 2.0 fluorometric assay (Life Technologies, Inc., Darmstadt, Germany) and analyzed with an Agilent Bioanalyzer.

MLVA was performed with an ABI 3130 Capillary Sequencer as described previously (6, 8, 17). For *in silico* MLVA, in-house python scripts were used to determine the corresponding fragment lengths by using the published primer sets.

NGS was performed by the Illumina MiSeq approach. In accordance with the manufacturer’s protocol, sequencing libraries were prepared by using 2.0 ng of genomic DNA with the Nextera XT kit (Illumina, San Diego, CA). Genomes were sequenced on an Illumina MiSeq platform with paired-end v 3 chemistry (2 × 300 bp). *De novo* genome assembly was performed with SPAdes (18) version 3.5.0 and polished by Pilon v.1.3.0 (19).

For SNP identification, the Northern Arizona SNP pipeline was used (http://tgennorth.github.io/NASP/) (16). This includes the alignment of raw data against the Ames ancestor (NC_007530, NC_007322, and NC_007323) with the BWA-MEM algorithm (20) and SNP calling by the UnifiedGenotyper method in GATK (21, 22). To calculate the depth of coverage, raw data were aligned with the Ames ancestor and the per-base depth of coverage was calculated by the GenomeCoverageBed method in BEDTools (23).

### Accession number(s).

Sequence reads were deposited at the NCBI Short Read Archive under accession no. SRR5275585 (sugar 2), SRR5275584 (sugar 3), and SRR5275583 (sugar 4).

## References

[1] Arias A, Watson SJ, Asogun D, Tobin EA, Lu J, Phan MVT, Jah U, Wadoum REG, Meredith L, Thorne L, Caddy S, Tarawalie A, Langat P, Dudas G, Faria NR, Dellicour S, Kamara A, Kargbo B, Kamara BO, Gevao S, Cooper D, Newport M, Horby P, Dunning J, Sahr F, Brooks T, Simpson AJH, Groppelli E, Liu G, Mulakken N, Rhodes K, Akpablie J, Yoti Z, Lamunu M, Vitto E, Otim P, Owilli C, Boateng I, Okoror L, Omomoh E, Oyakhilome J, Omiunu R, Yemisis I, Adomeh D, Ehikhiametalor S, Akhilomen P, Aire C, Kurth A, Cook N, Baumann J, et al. 2016 Rapid outbreak sequencing of Ebola virus in Sierra Leone identifies transmission chains linked to sporadic cases. Virus Evol 2:vew016. doi:10.1093/ve/vew016. PMC5499387 28694998

[2] Wagner DM, Klunk J, Harbeck M, Devault A, Waglechner N, Sahl JW, Enk J, Birdsell DN, Kuch M, Lumibao C, Poinar D, Pearson T, Fourment M, Golding B, Riehm JM, Earn DJD, DeWitte S, Rouillard JM, Grupe G, Wiechmann I, Bliska JB, Keim PS, Scholz HC, Holmes EC, Poinar H 2014 Yersinia pestis and the plague of Justinian 541–543 AD: a genomic analysis. Lancet Infect Dis 14:319–326. doi:10.1016/S1473-3099(13)70323-2. 24480148

[3] Duggan AT, Perdomo MF, Piombino-Mascali D, Marciniak S, Poinar D, Emery MV, Buchmann JP, Duchêne S, Jankauskas R, Humphreys M, Golding GB, Southon J, Devault A, Rouillard JM, Sahl JW, Dutour O, Hedman K, Sajantila A, Smith GL, Holmes EC, Poinar HN 2016 17th century variola virus reveals the recent history of smallpox. Curr Biol 26:3407–3412. doi:10.1016/j.cub.2016.10.061. 27939314 PMC5196022

[4] Redmond C, Pearce MJ, Manchee RJ, Berdal BP 1998 Deadly relic of the Great War. Nature 393:747–748. doi:10.1038/31612. 9655389

[5] Søhr J 1938 Spioner og bomber. Fra opdagelsespolitiets arbeide under verdenskrigen, p 35–46. Johan Grundt Tanum, Oslo, Norway (In Norwegian.)

[6] Van Ert MN, Easterday WR, Huynh LY, Okinaka RT, Hugh-Jones ME, Ravel J, Zanecki SR, Pearson T, Simonson TS, U’Ren JM, Kachur SM, Leadem-Dougherty RR, Rhoton SD, Zinser G, Farlow J, Coker PR, Smith KL, Wang B, Kenefic LJ, Fraser-Liggett CM, Wagner DM, Keim P 2007 Global genetic population structure of Bacillus anthracis. PLoS One 2:e461. doi:10.1371/journal.pone.0000461. 17520020 PMC1866244

[7] Okinaka RT, Henrie M, Hill KK, Lowery KS, Van Ert M, Pearson T, Schupp J, Kenefic L, Beaudry J, Hofstadler SA, Jackson PJ, Keim P 2008 Single nucleotide polymorphism typing of Bacillus anthracis from Sverdlovsk tissue. Emerg Infect Dis 14:653–656. doi:10.3201/eid1404.070984. 18394287 PMC2570946

[8] Lista F, Faggioni G, Valjevac S, Ciammaruconi A, Vaissaire J, le Doujet C, Gorgé O, De Santis R, Carattoli A, Ciervo A, Fasanella A, Orsini F, D’Amelio R, Pourcel C, Cassone A, Vergnaud G 2006 Genotyping of Bacillus anthracis strains based on automated capillary 25-loci multiple locus variable-number tandem repeats analysis. BMC Microbiol 6:33. doi:10.1186/1471-2180-6-33. 16600037 PMC1479350

[9] Ravel J, Jiang L, Stanley ST, Wilson MR, Decker RS, Read TD, Worsham P, Keim PS, Salzberg SL, Fraser-Liggett CM, Rasko DA 2009 The complete genome sequence of Bacillus anthracis Ames “ancestor.” J Bacteriol 191:445–446. doi:10.1128/JB.01347-08. 18952800 PMC2612425

[10] Kenefic LJ, Pearson T, Okinaka RT, Schupp JM, Wagner DM, Hoffmaster AR, Trim CB, Trim CP, Chung WK, Beaudry JA, Jiang L, Gajer P, Foster JT, Mead JI, Ravel J, Keim P 2009 Pre-Columbian origins for North American anthrax. PLoS One 4:e4813. doi:10.1371/journal.pone.0004813. 19283072 PMC2653229

[11] Simonson TS, Okinaka RT, Wang B, Easterday WR, Huynh L, U’Ren JM, Dukerich M, Zanecki SR, Kenefic LJ, Beaudry J, Schupp JM, Pearson T, Wagner DM, Hoffmaster A, Ravel J, Keim P 2009 Bacillus anthracis in China and its relationship to worldwide lineages. BMC Microbiol 9:71. doi:10.1186/1471-2180-9-71. 19368722 PMC2674057

[12] Read TD, Salzberg SL, Pop M, Shumway M, Umayam L, Jiang L, Holtzapple E, Busch JD, Smith KL, Schupp JM, Solomon D, Keim P, Fraser CM 2002 Comparative genome sequencing for discovery of novel polymorphisms in Bacillus anthracis. Science 296:2028–2033. doi:10.1126/science.1071837. 12004073

[13] Read TD, Peterson SN, Friedlander AM, Thomason B, Salzberg SL, White O, Thwaite JE, Redmond C, Cline R, Hazen A, Nierman WC, Watkins KL, Wolf AM, Plaut RD, Berry KJ, Weidman JF, Hance IR, Jiang L, Mahamoud Y, Benton JL, Radune D, Khouri HM, Pop M, Peterson JD, Nelson WC, Haft DH, Durkin AS, Daugherty SC, Madpu R, DeBoy RT, Gwinn M, Brinkac LM, Dodson RJ, Beanan MJ, Kolonay JF, Wu M, Rilstone J, Helgason E, Økstad OA, Holtzapple EK, Gill SR, Eisen JA, Fouts DE, Tettelin H, Nelson KE, Paulsen IT, Baillie LW, Tourasse N, Friedlander AM, Koehler TM, et al. 2003 The genome sequence of Bacillus anthracis Ames and comparison to closely related bacteria. Nature 423:81–86. doi:10.1038/nature01586. 12721629

[14] Sue D, Marston CK, Hoffmaster AR, Wilkins PP 2007 Genetic diversity in a Bacillus anthracis historical collection (1954 to 1988). J Clin Microbiol 45:1777–1782. doi:10.1128/JCM.02488-06. 17392445 PMC1933066

[15] Keim P, Price LB, Klevytska AM, Smith KL, Schupp JM, Okinaka R, Jackson PJ, Hugh-Jones ME 2000 Multiple-locus variable-number tandem repeat analysis reveals genetic relationships within Bacillus anthracis. J Bacteriol 182:2928–2936. doi:10.1128/JB.182.10.2928-2936.2000. 10781564 PMC102004

[16] Sahl JW, Pearson T, Okinaka R, Schupp JM, Gillece JD, Heaton H, Birdsell D, Hepp C, Fofanov V, Noseda R, Fasanella A, Hoffmaster A, Wagner DM, Keim P 2016 A Bacillus anthracis genome sequence from the Sverdlovsk 1979 autopsy specimens. mBio 7. doi:10.1128/mBio.01501-16. PMC5050339 27677796

[17] Antwerpen M, Ilin D, Georgieva E, Meyer H, Savov E, Frangoulidis D 2011 MLVA and SNP analysis identified a unique genetic cluster in Bulgarian Bacillus anthracis strains. Eur J Clin Microbiol Infect Dis 30:923–930. doi:10.1007/s10096-011-1177-2. 21279731

[18] Bankevich A, Nurk S, Antipov D, Gurevich AA, Dvorkin M, Kulikov AS, Lesin VM, Nikolenko SI, Pham S, Prjibelski AD, Pyshkin AV, Sirotkin AV, Vyahhi N, Tesler G, Alekseyev MA, Pevzner PA 2012 SPAdes: a new genome assembly algorithm and its applications to single-cell sequencing. J Comput Biol 19:455–477. doi:10.1089/cmb.2012.0021. 22506599 PMC3342519

[19] Walker BJ, Abeel T, Shea T, Priest M, Abouelliel A, Sakthikumar S, Cuomo CA, Zeng Q, Wortman J, Young SK, Earl AM 2014 Pilon: an integrated tool for comprehensive microbial variant detection and genome assembly improvement. PLoS One 9:e112963. doi:10.1371/journal.pone.0112963. 25409509 PMC4237348

[20] Li H 26 5 2013 Aligning sequence reads, clone sequences and assembly contigs with BWA-MEM. arXiv arXiv:1303 3997 [q-bio.GN] https://arxiv.org/abs/1303.3997.

[21] DePristo MA, Banks E, Poplin R, Garimella KV, Maguire JR, Hartl C, Philippakis AA, del Angel G, Rivas MA, Hanna M, McKenna A, Fennell TJ, Kernytsky AM, Sivachenko AY, Cibulskis K, Gabriel SB, Altshuler D, Daly MJ 2011 A framework for variation discovery and genotyping using next-generation DNA sequencing data. Nat Genet 43:491–498. doi:10.1038/ng.806. 21478889 PMC3083463

[22] McKenna A, Hanna M, Banks E, Sivachenko A, Cibulskis K, Kernytsky A, Garimella K, Altshuler D, Gabriel S, Daly M, DePristo MA 2010 The genome analysis toolkit: a MapReduce framework for analyzing next-generation DNA sequencing data. Genome Res 20:1297–1303. doi:10.1101/gr.107524.110. 20644199 PMC2928508

[23] Quinlan AR, Hall IM 2010 BEDTools: a flexible suite of utilities for comparing genomic features. Bioinformatics 26:841–842. doi:10.1093/bioinformatics/btq033. 20110278 PMC2832824

